# The Refraction of the Eye, Including a Complete Treatise on Ophthalmometry

**Published:** 1900-08

**Authors:** 


THE REFRACTION OF THE EYE, INCLUDING A COMPLETE TREATISE ON OPHTHALMOMETRY. A Clinical Text-book for Students and Practitioners. By EDWARD DAVIS, A. M., M. D., Adjunct Professor of the Eye in the New York Post-Graduate Medical School and Hospital; Assistant Surgeon to the Manhattan Eye and Ear Hospital; Attending Ophthalmic Surgeon to the Babies' Wards of the New York Post-Graduate Hospital; Attending Ophthalmic Surgeon to Bellevue Hospital Out-Door Department; Secretary of the New York Ophthalmological Society; Assistant Secretary of the New York Physicians' Mutual Aid Association; Member of the New York State Medical Society, County Medical Society, Academy of Medicine, etc. With 190 engravings, 97 of which are original. Duodecimo, pp. xiv.—432. New York and London: The MacMillan Company. 1900. [Price, cloth, $3.00.]

Dr. Davis has here written especially for students. His work embraces a full description of the ophthalmometer and gives detailed instruction as to using it. The author also describes, minutely, his methods of determining errors of refraction, and cites 150 cases in illustration. The description of the ophthalmometer is excellent, and the methods of estimating and correcting errors of refraction are in the main as serviceable as any that can be adopted without using a cycloplegic. A few illustrative cases are often helpful, but the utility of so large a number as are here introduced is questionable, even with the aid of the author's index to them.

Throughout the book, the author has endeavored "to show the utter uselessness of a mydriatic in fitting glasses in a vast majority of cases, even in young subjects," and in the measurement of astigmatism he relies largely on the readings of the ophthalmometer. In both his hospital and private practice he does not find it necessary to "use a mydriatic in more than 1 per cent. of all cases of refraction," urging that "when we take into account the time and great annoyance saved to the patient by not using atropine, we can readily see the advantages of the ophthalmometer." But many painstaking and clear-headed observers have demonstrated repeatedly that the readings of the ophthalmometer are, at the best, only suggestive, and that the final subjective findings show, altogether too frequently, a marked variation both in the degree of astigmatism and in the direction of its axis—a variation that counts seriously against the power to estimate the total ocular astigmatism by ascertaining the corneal astigmatism by means of any instrument whatsoever. As to the use of atropine, the author seems to assume that this is the only available cycloplegic; and if it were, others would readily join him in discouraging its use. But with other cycloplegics at hand, whose disabling effects are so transient, his objection to "a mydriatic" has but little weight. Most ophthalmologists use atropine very rarely in refractive work, as homatropine is generally all-sufficient. The duration of its effects is brief, it quite fully relaxes the accommodation, an examination is made much more expeditiously, the errors of refraction are determined at once and with greater certainty, the patient is saved the time by making several visits and the ophthalmologist is far more sure of a diagnosis by which he is guided to the proper treatment.

The author's teachings are summarised on page 41 as follows: "(1) Use the ophthalmometer; (2) Use trial lenses and test cards; (3) Use the ophthalmoscope; (4) If after two tests on different days the result is still unsatisfactory, employ a mydriatic and use the retinoscope in addition to the other tests."

Those who would inform themselves in regard to the views and methods of those who plead for the reliability of the ophthalmometer, and who try to belittle the need and helpfulness of a cycloplegic in determining errors of refraction in subjects with active accommodation, may read this book; but it is an unsafe guide to those who desire to learn how to do refraction work with expedition and accuracy.

